# Supplementary material for: Glucose-regulated and drug-perturbed phosphoproteome reveals molecular mechanisms controlling insulin secretion
Source: Nat Commun. 2016 Nov 14;7:13250. doi: 10.1038/ncomms13250 (PMC5114537; doi:10.1038/ncomms13250)
Supplement: Supplementary Information — Supplementary Figures 1 - 11, Supplementary Methods 1 and Supplementary References [file ncomms13250-s1.pdf]

Supplementary material for Sacco et al.

Supplementary Figures

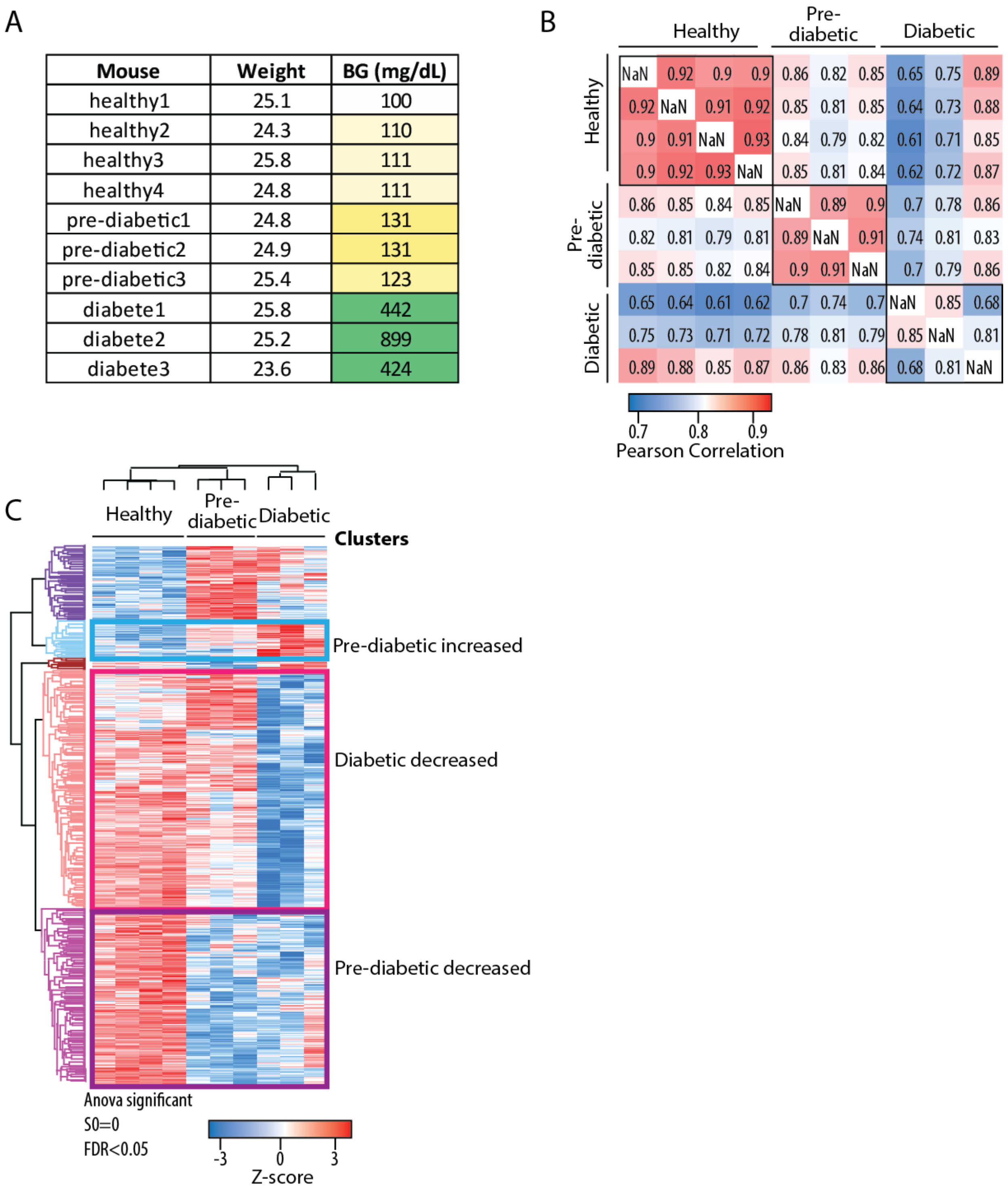

Mouse	Weight	BG (mg/dL)
healthy1	25.1	100
healthy2	24.3	110
healthy3	25.8	111
healthy4	24.8	111
pre-diabetic1	24.8	131
pre-diabetic2	24.9	131
pre-diabetic3	25.4	123
diabete1	25.8	442
diabete2	25.2	899
diabete3	23.6	424

Supplementary Figure 1. Data summary for the NOD islets proteomics dataset. A) Weight and blood glucose concentrations for each mouse. **B**) Heat map showing the Pearson correlation coefficients between the different biological replicates. **C**) Unsupervised hierarchical clustering representing the ANOVA significant proteins (Log2 LFQ intensity is Z-scored).

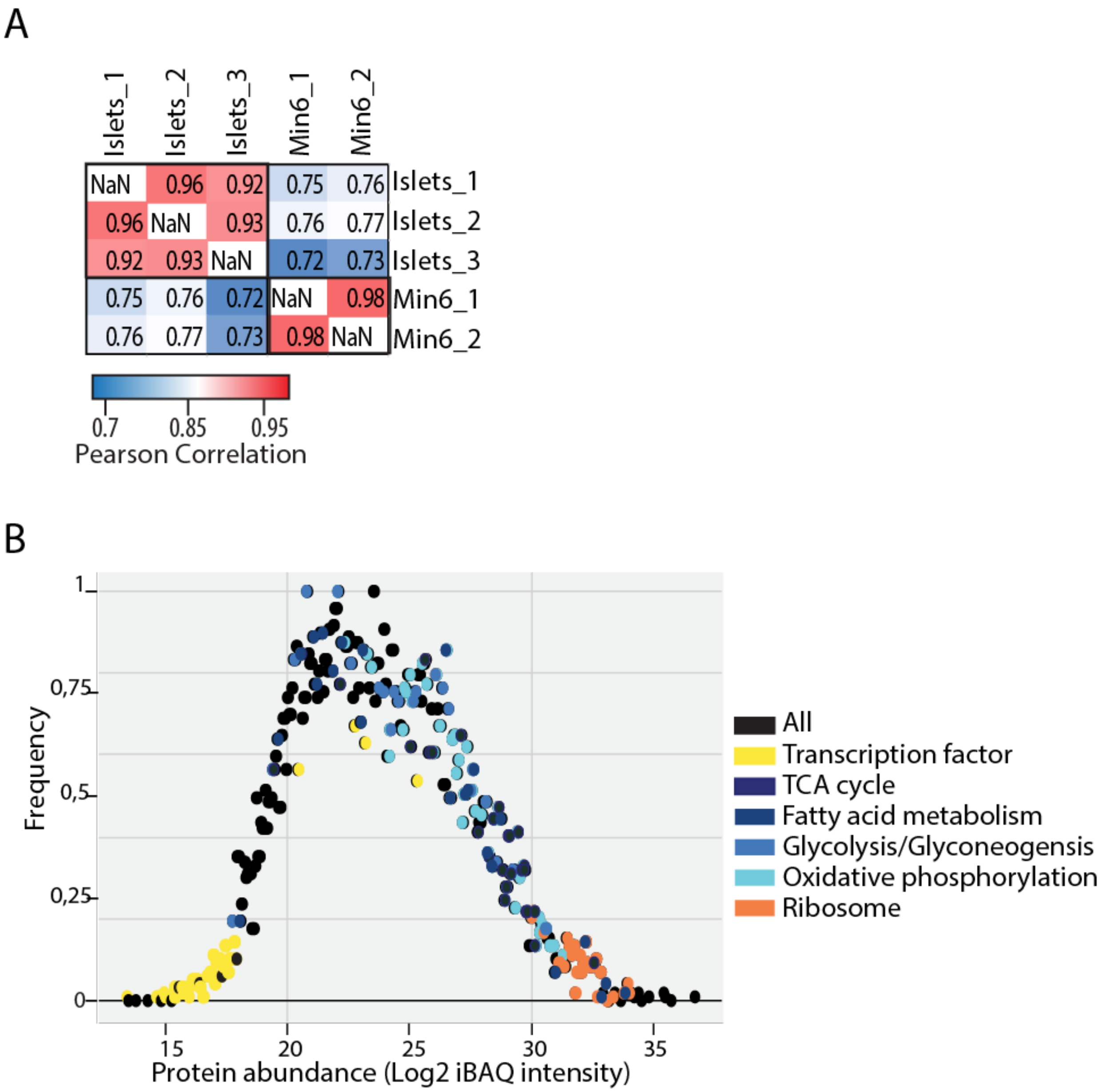

Supplementary Figure 2. Data summary for the proteomics dataset. A) Heat map showing the Pearson correlation coefficients between the different biological replicates. **B**) Distribution of protein abundances. Proteins involved in certain biological processes (GO-BP) are highlighted in different color, as indicated in the legend.

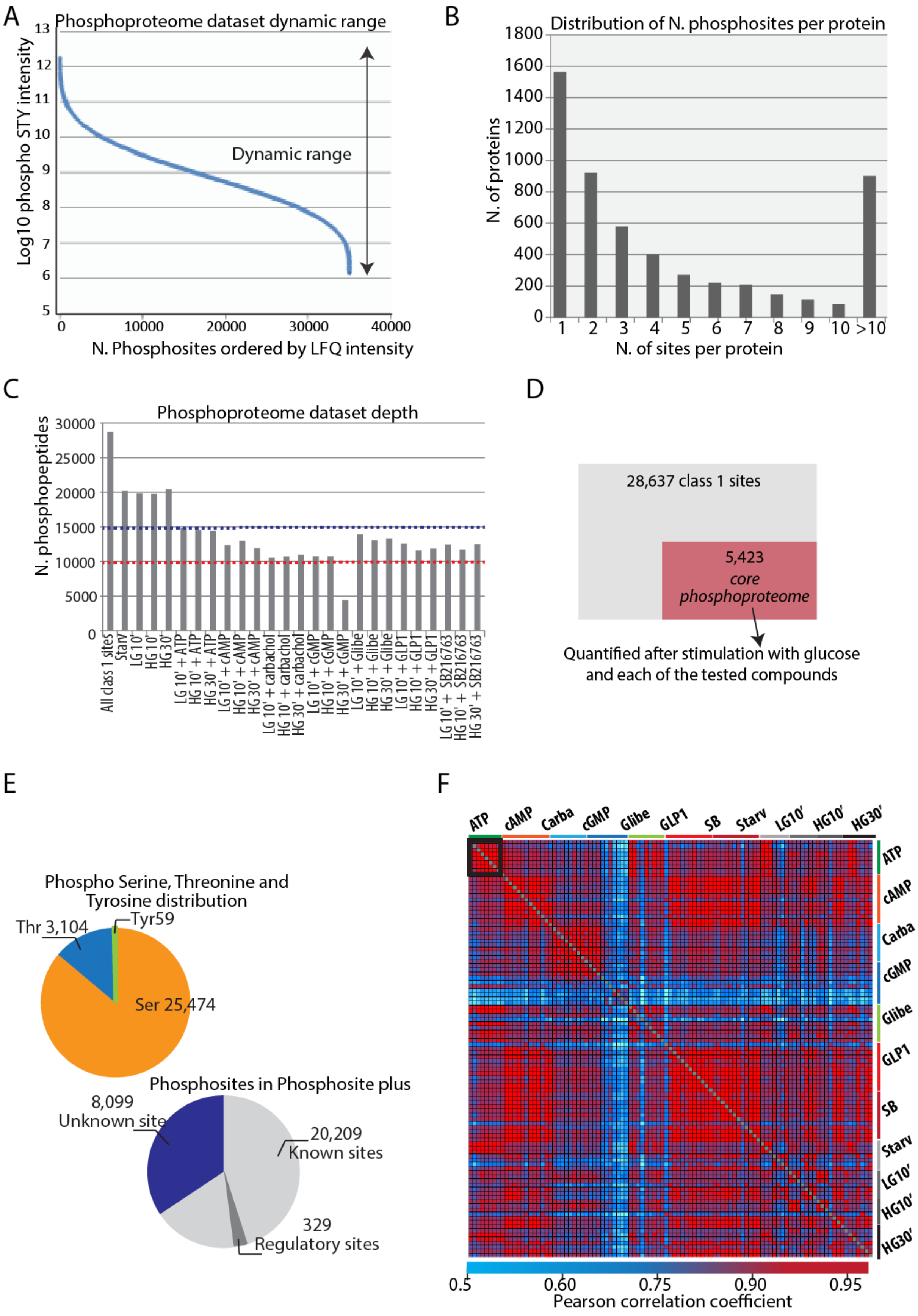
A
Phosphoproteome dataset dynamic range
Log10 phospho STY intensity
Dynamic range
N. Phosphosites ordered by LFQ intensity
B
Distribution of N. phosphosites per protein
N. of proteins
N. of sites per protein
C
Phosphoproteome dataset depth
N. phosphopeptides
All class 1 sites
Starv
LG 10'
HG 10'
HG 30'
LG 10' + ATP
HG 10' + ATP
HG 30' + ATP
LG 10' + cAMP
HG 10' + cAMP
HG 30' + cAMP
LG 10' + carbachol
HG 10' + carbachol
HG 30' + carbachol
LG 10' + cGMP
HG 10' + cGMP
HG 30' + cGMP
LG 10' + Glibe
HG 10' + Glibe
HG 30' + Glibe
LG 10' + GLP1
HG 10' + GLP1
HG 30' + GLP1
LG 10' + SB216763
HG 10' + SB216763
HG 30' + SB216763
D
28,637 class 1 sites
5,423
core
phosphoproteome
Quantified after stimulation with glucose
and each of the tested compounds
E
Phospho Serine, Threonine and
Tyrosine distribution
Thr 3,104
Tyr59
Ser 25,474
Phosphosites in Phosphosite plus
8,099
Unknown site
20,209
Known sites
329
Regulatory sites
F
ATP
cAMP
Carba
cGMP
Glibe
GLP1
SB
Starv
LG10'
HG10'
HG30'
Pearson correlation coefficient

Supplementary Figure 3. Data summary for the phosphoproteomics dataset. A) Dynamic range of the phosphosites of measured samples, based on their Log10 label-free intensities (LFQ). **B)** Distribution of number of phosphosites per protein. **C)** Number of phosphopeptides per experimental condition. **D)** Quantification coverage of unique phosphopeptide species (singly, doubly or greater phosphopeptides) containing only localized (Class 1) phosphosites. Inner circle denotes phosphopeptides quantified in all drug and glucose stimulated conditions (core phosphoproteome). **E**) In the upper part, the distribution of serine, threonine and tyrosine phosphorylation sites is quantified. The lower part shows phosphosites quantified in our study that are already present in phosphositeplus and annotated as “regulatory sites”. **F)** Heat map showing the Pearson correlation coefficient between the different biological replicates.

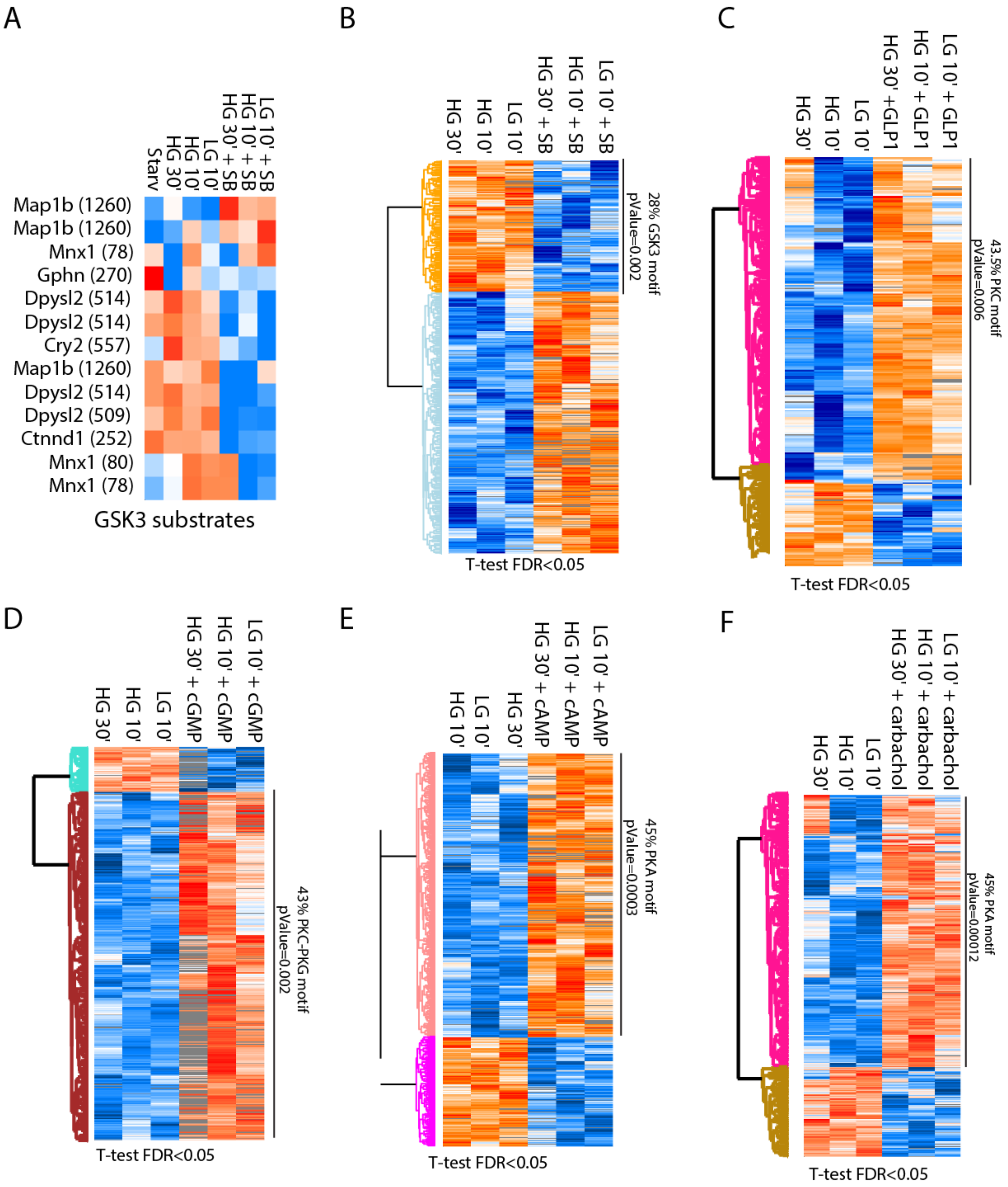

Supplementary Figure 4. Drugs efficiently target their direct substrates. A) GSK3-specific substrates were extracted from our phosphoproteome dataset and their phosphorylation status is represented as a heatmap. Unsupervised hierarchical clustering representing the T test significant

phosphosites between the SB216763 (**B**) or GLP1 (**C**) or cGMP (**D**) or cAMP (**E**) or carbachol (**F**) and glucose treated beta cells.

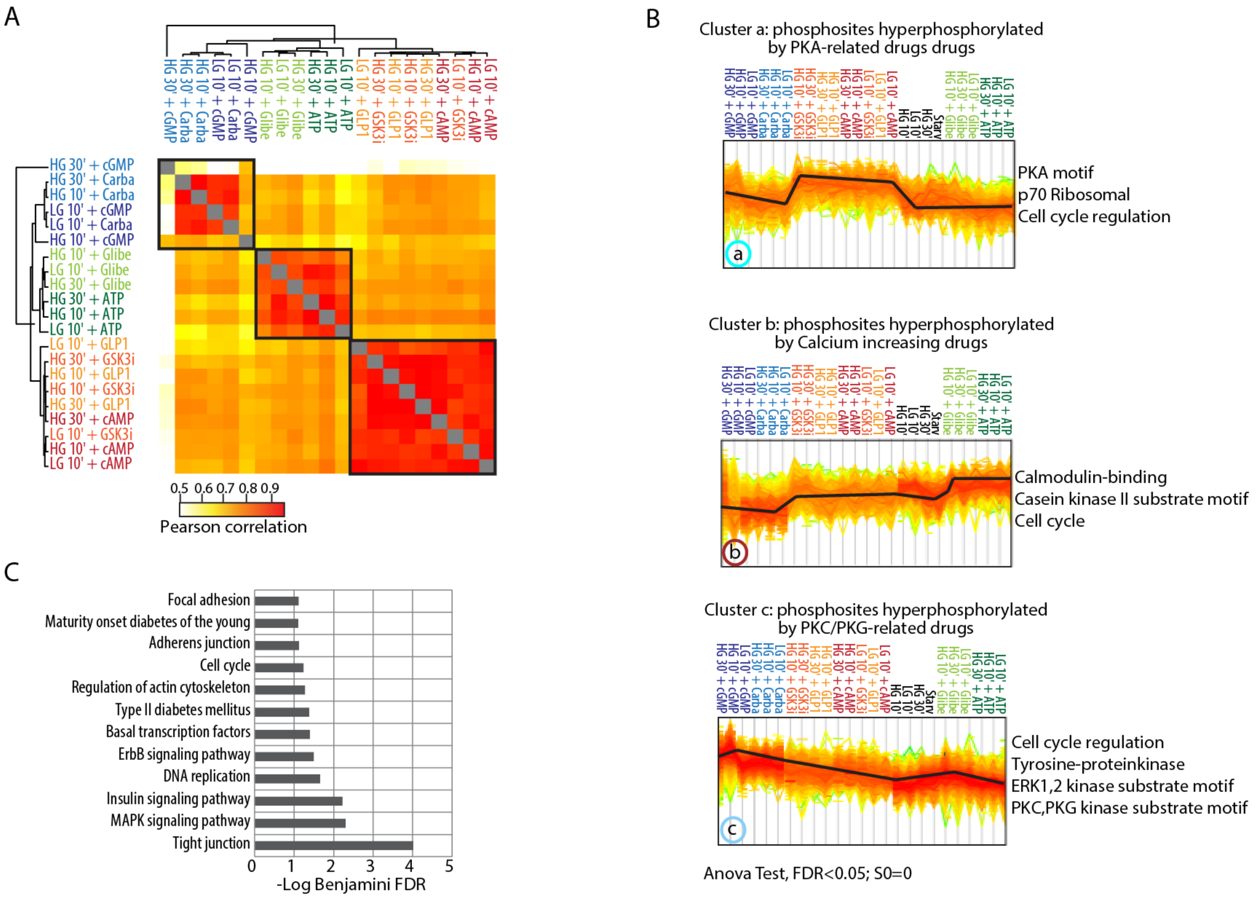

Supplementary Figure 5. Classification of drugs in three different groups according to the downstream kinase activated. A) Unsupervised hierarchical clustering of Pearson correlation coefficients between each of the experimental conditions. **B)** Pathways and GO-Biological processes significantly enriched in three representative clusters are shown. **C)** 1D annotation analysis of the ANOVA significant phosphoproteins.

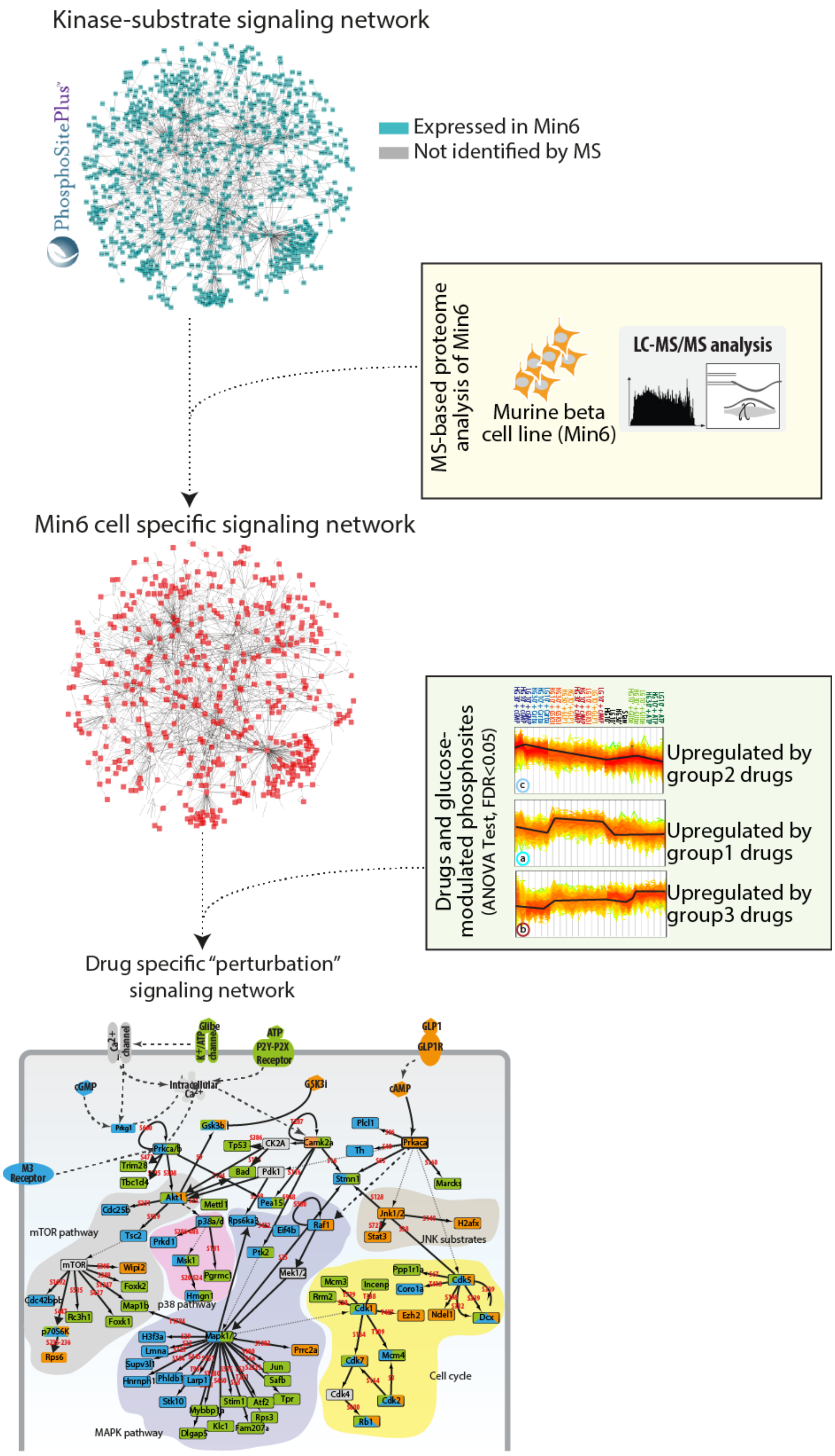
Kinase-substrate signaling network
PhosphoSitePlus
Expressed in Min6
Not identified by MS
MS-based proteome analysis of Min6
Murine beta cell line (Min6)
LC-MS/MS analysis
Min6 cell specific signaling network
Drugs and glucose-modulated phosphosites (ANOVA Test, FDR<0.05)
Upregulated by group2 drugs
Upregulated by group1 drugs
Upregulated by group3 drugs
Drug specific "perturbation" signaling network
mTOR pathway
p38 pathway
JNK substrates
Cell cycle
MAPK pathway

Supplementary Figure 6. Mapping drug effects on signaling network. Global naïve network of signaling information retrieved from PhosphositePlus database (upper part). Min6 beta cell specific signaling network was filtered to keep only relationships between proteins that were found to be expressed in our proteomic analysis (lower part). Phosphoproteomics data were overlaid and the resulting network was filtered according [7].

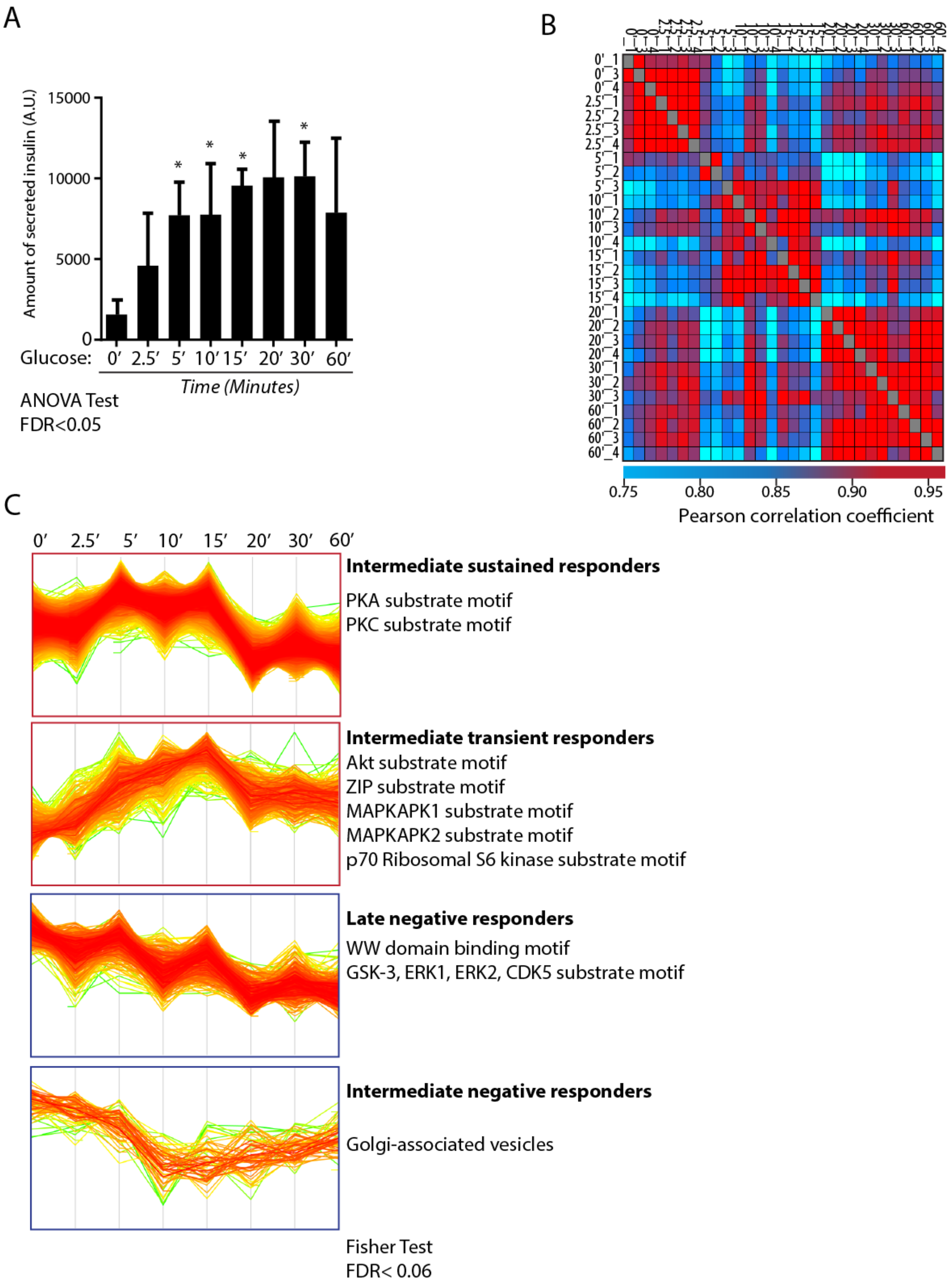
A
Amount of secreted insulin (A.U.)
15000
10000
5000
0
Glucose:
0′ 2.5′ 5′ 10′ 15′ 20′ 30′ 60′
Time (Minutes)
ANOVA Test
FDR<0.05
B
0'_1
0'_3
0'_4
2.5'_1
2.5'_2
2.5'_3
2.5'_4
5'_1
5'_2
5'_3
10'_1
10'_2
10'_3
10'_4
15'_1
15'_2
15'_3
15'_4
20'_1
20'_2
20'_3
20'_4
30'_1
30'_2
30'_3
60'_1
60'_2
60'_3
60'_4
0.75
0.80
0.85
0.90
0.95
Pearson correlation coefficient
C
0′ 2.5′ 5′ 10′ 15′ 20′ 30′ 60′
Intermediate sustained responders
PKA substrate motif
PKC substrate motif
Intermediate transient responders
Akt substrate motif
ZIP substrate motif
MAPKAPK1 substrate motif
MAPKAPK2 substrate motif
p70 Ribosomal S6 kinase substrate motif
Late negative responders
WW domain binding motif
GSK-3, ERK1, ERK2, CDK5 substrate motif
Intermediate negative responders
Golgi-associated vesicles
Fisher Test
FDR< 0.06

Supplementary Figure 7. Time-resolved analysis of phosphoproteome in beta cells upon glucose stimulation. A) Amount of secreted insulin after drug and glucose stimulation measured (Elisa assay) and shown as a bar graph (* pValue <0.05). Median of 4 biological replicates and standard deviation is plotted. **B**) Heat map showing the Pearson correlation coefficient between the different biological replicates. **C**) Kinase substrate motifs and GO-Biological processes significantly enriched in four representative clusters of significantly modulated phosphosites (ANOVA test, FDR<0.05) are shown.

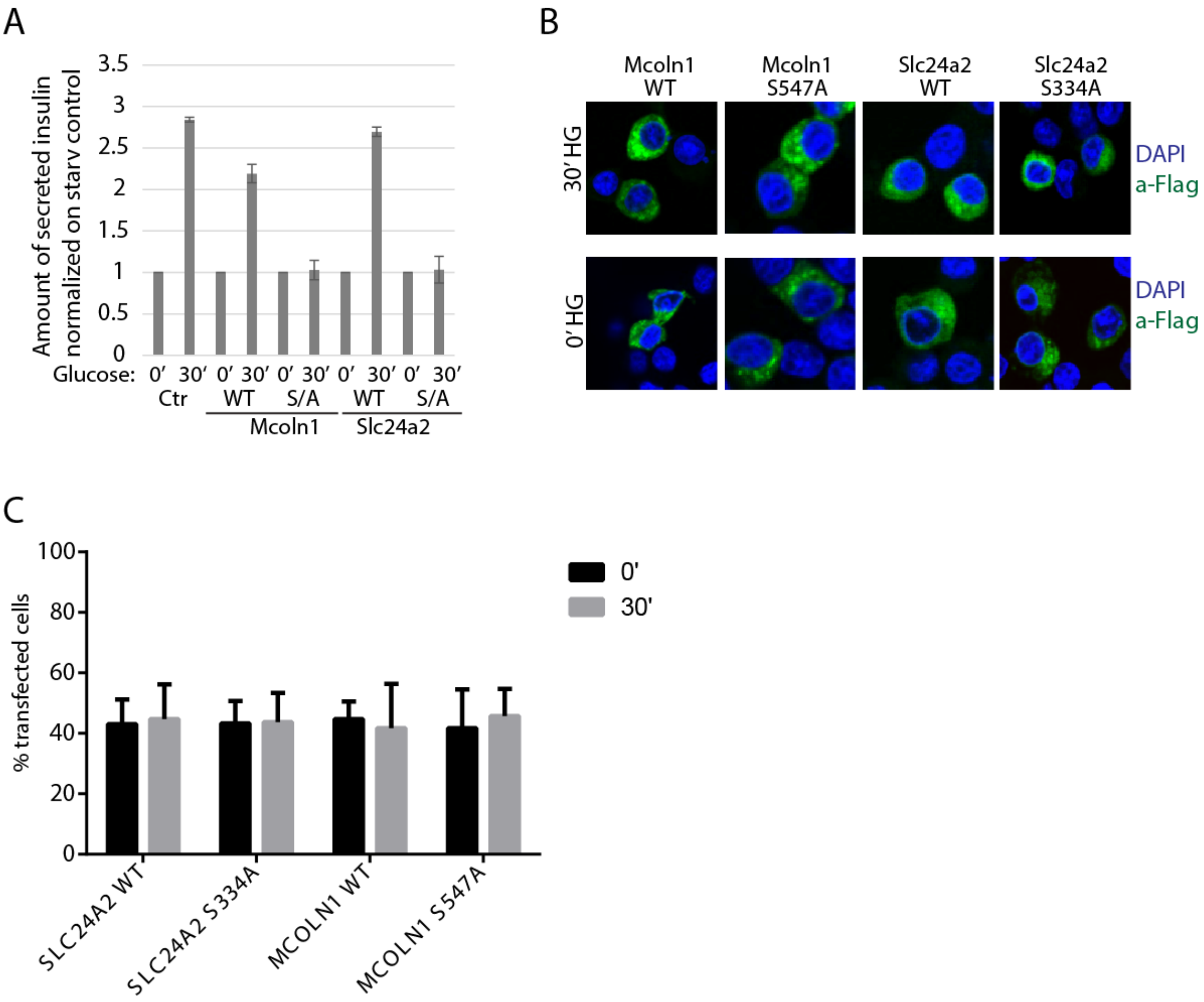

Supplementary Figure 8. The phosphorylation of Mcoln1 and Slc24a2 channels affects glucose-induced insulin secretion. A) Ins1e transiently transfected with the wild type form or the unphosphorylable (S547A of Mcoln1, S334A of Slc24a2) mutant of Mcoln1 or Slc24a2 or empty vector as negative control, were stimulated with high glucose for 30 minutes or left untreated. The amount of secreted insulin is shown as a bar graph. Each bar represents the median and standard deviation of three biological replicates normalized on the starvation control. **B**) Intracellular localization of the wild type form or the unphosphorylable (serine to alanine substitution) mutant of Mcoln1 or Slc24a2 by indirect immunofluorescence microscopy. **C**) The

number of Flag fluorescent cells was quantified on at least 100 cells per condition, using the Cell Profiler software. Results are shown as median of the percentage of Flag-positive cells. Standard deviation is reported.

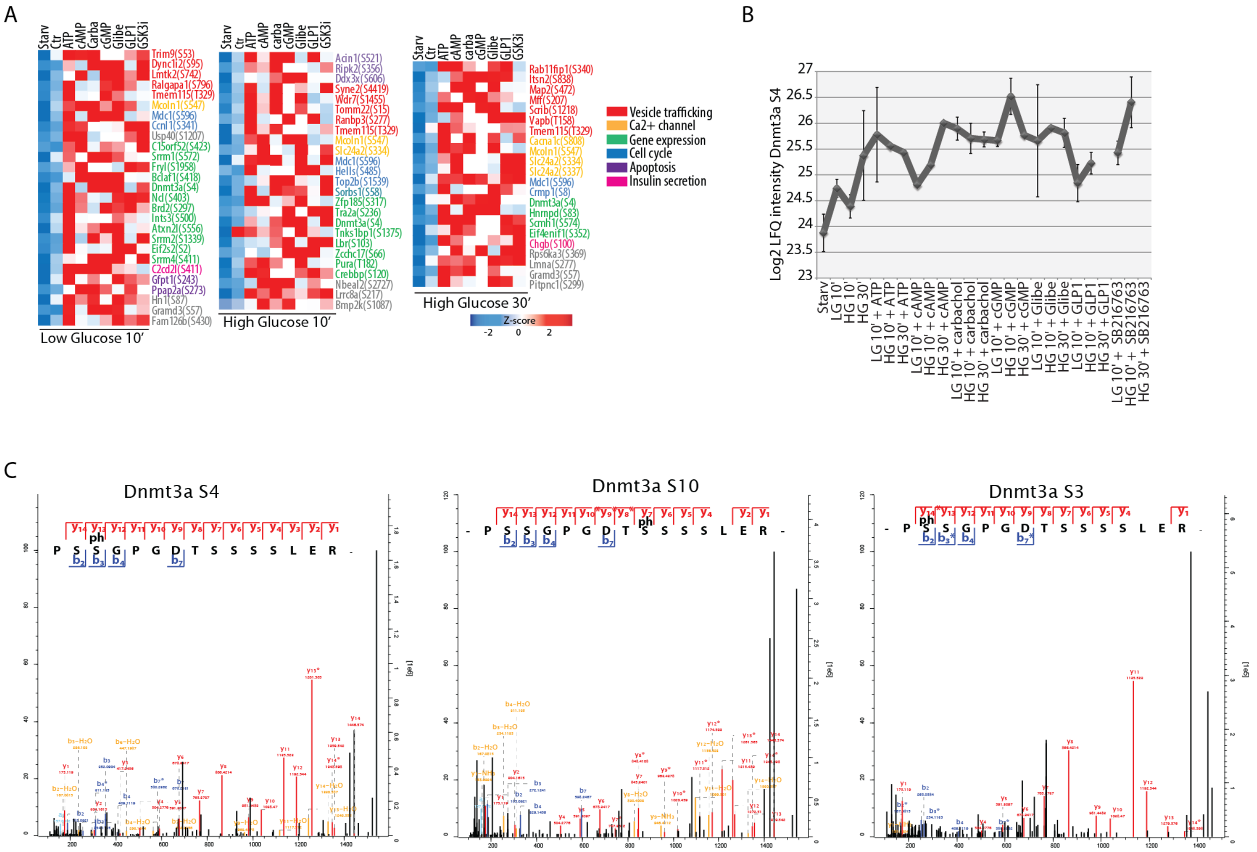

Supplementary Figure 9. DNMT3A S7 phosphorylation is stimulated after glucose stimulation and further increased after drug treatment. A) Heat map of the novel regulatory sites phosphorylated after glucose and drug stimulation. **B**) Median intensity of the Dnmt3a S7 phosphorylation in beta cells glucose and drug stimulated. SEM of at least triplicates are reported. **C**) HCD MS/MS spectra of the pS3-Dnmt3a, pS4-Dnmt3a and pS10-Dnmt3a peptides identified in our drug and glucose phosphoproteome screening.

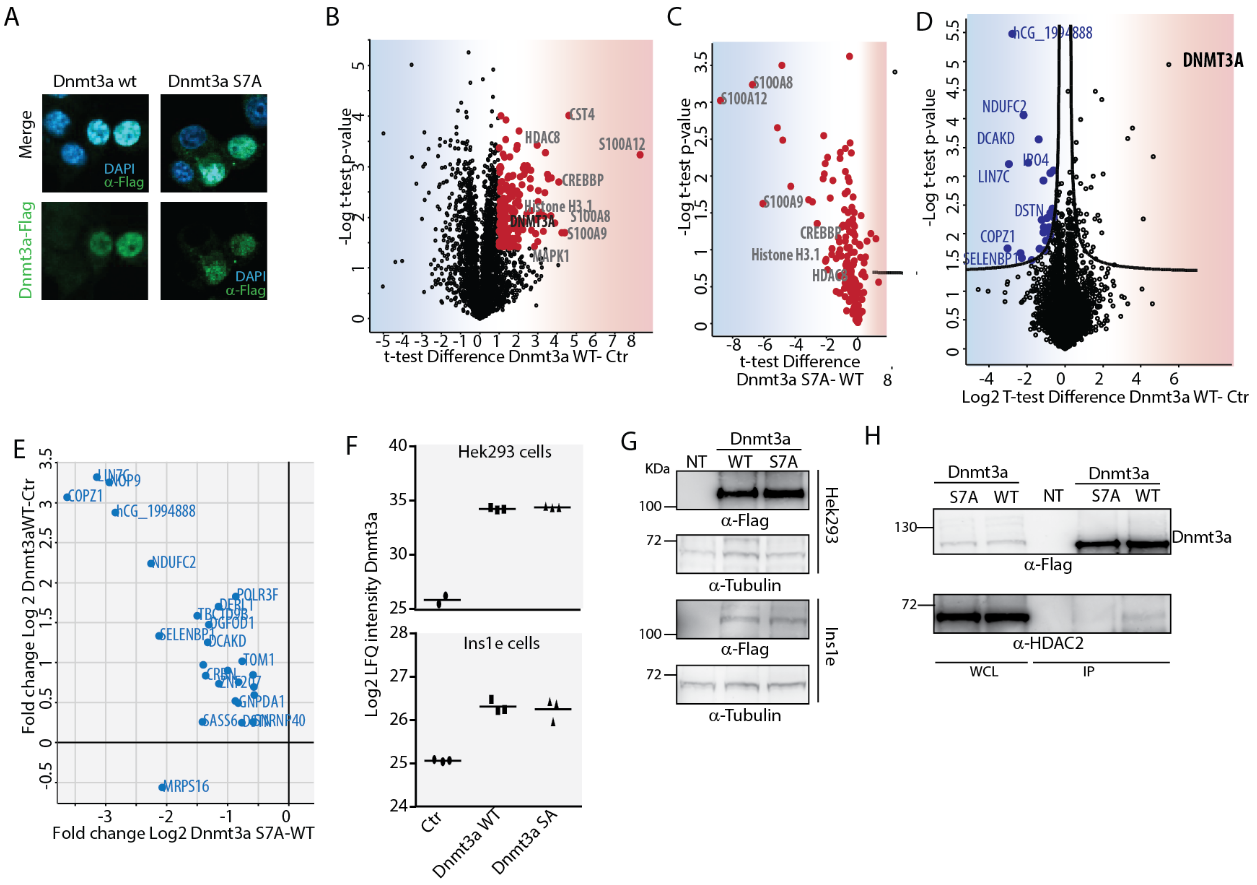

Supplementary Figure 10. Dnmt3a S7 phosphorylation controls the methyltransferase interactome without affecting its localization. A) Intracellular localization of the wild type form or the unphosphorylable (S7A) mutant of DNMT3A by indirect immunofluorescence microscopy. **B**) Volcano plot of MS-quantified wild-type DNMT3A interactors in Hek293. **C**) Volcano plot of MS-quantified proteins in wild type and S7A mutant in Hek293 cells. **D**) Volcano plot of MS-quantified proteins after the over-expression of wild type form of Dnmt3a or empty vector, as control, in Hek293 cells. **E**) Scatterplot of significantly down-regulated proteins after the over-expression of wild type or S7A mutant forms of DNMT3A in Hek293 cells. **F**) Scatterplot of DNMT3A abundance (Log2 LFQ intensity) in cells over-expressing the wild type or S7A mutant forms of Dnmt3a or empty vector (Ctr), as negative control. **G**) Western blots illustrating the WT and S7A Dnmt3a-Flag over-expressed proteins in HEK293 and Ins1e cell

lines. H) WT and S7A forms of Dnmt3a were over-expressed in HEK293 cells, lysates subjected to immunoblot analysis or immunoprecipitation using α-FLAGand anti-HDAC2 antibodies.

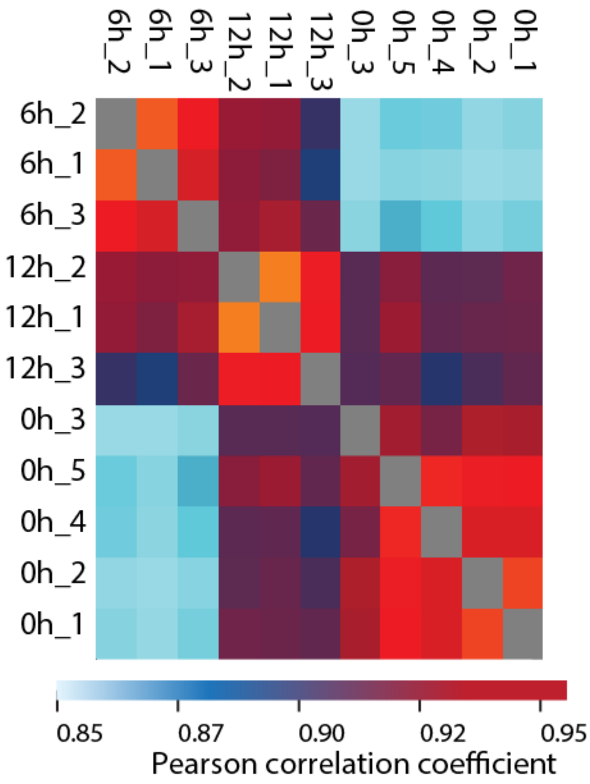

Supplementary Figure 11. Correlation between proteomes of glucose-stimulated beta cells. Heat map showing the Pearson correlation coefficients between the different biological replicates is reported.

Supplementary methods

Proteome and phosphoproteome data processing

Raw mass spectrometry data were analyzed in the MaxQuant environment [1], version 1.5.0.27, employing the Andromeda engine for database search[2]. MS/MS spectra were matched against the Mus Murine UniProt FASTA database (September 2014), with an FDR of <1% at the level of proteins, peptides and modifications. Enzyme specificity was set to trypsin, allowing for cleavage N-terminal to proline and between aspartic acid and proline. The search included cysteine carbamidomethylation as a fixed modification, and N-terminal protein acetylation, oxidation of methionine and phosphorylation of serine, threonine tyrosine residue (STY) as variable modifications. Label free proteome analysis was performed in MaxQuant[3]. For proteome and phosphoproteome analysis, where possible, the identity of peptides present but not sequenced in a given run was obtained by transferring identifications across liquid chromatography (LC)-MS runs ('match between runs') [1, 4]. For phosphopeptide identification, an Andromeda minimum score and minimum delta score threshold of 40 and 17 were used, respectively. Up to three missed cleavages were allowed for protease digestion and peptides had to be fully tryptic.

Proteome and phosphoproteome bioinformatics data analysis

Bioinformatic analysis was performed in the Perseus software environment [5]. Statistical analysis of proteome, phosphoproteome and immunoprecipitations was performed on logarithmized intensities for those values that were found to be quantified in any experimental condition. To identify significantly modulated phosphopetides across many different conditions, we performed an ANOVA analysis of grouped biological replicates, with a permutation-based FDR cutoff of

0.05. To identify significantly modulated proteins between two samples we performed *t*-test analysis of grouped biological replicates, with a permutation-based FDR cutoff of 0.05. Categorical annotation was added in Perseus in the form of GO biological process (GOBP), molecular function (GOMF), and cellular component (GOCC), KEGG pathways and kinase substrate motifs (extracted from HPRD). Concerning the kinase substrate motifs, we performed two analyses: (i) Fisher exact tests of the loadings (phosphosites) responsible for the discrimination of the three groups (Fig. 3B); (ii) 1D annotation enrichment analyses[6] to identify statistically significant enriched kinase-substrates motifs in the various experimental conditions (Fig. 4A and S3). Multiple hypothesis testing was controlled by using a Benjamini-Hochberg FDR threshold of 0.05. To analyze the enrichment in the kinase substrate motifs, we employed 1D annotation enrichment. In case of the kinase-substrate motif category it essentially tests whether the corresponding expression values have a preference to be systematically larger or smaller than the global distribution of expression values. Then for each kinase-substrate motif the corresponding pValue and score are assigned. While a score near 1 indicates a positive enrichment, a score near -1 means a negative enrichment of the category.

Combining proteome and phosphoproteome data with a prior knowledge signaling network

This strategy has been previously developed by our group [7]. Kinase-substrate relationships were extracted by PhosphositePlus (Kinase-substrate dataset) [8] and were mapped onto the complete human proteome. Then the network was first filtered to maintain only relationships between proteins that were identified in our proteomic analysis of Min6 cells. This Min6 specific network was used as a scaffold to overlay the changes at the phosphoproteome level induced by drug treatments (Fig. 3B). Next the network was filtered according to the following rules: i) “leaf

nodes” (connected only by one edge) whose phosphorylation was not quantified in our phospho-proteomics approach were excluded; ii) “Leaf nodes” whose phosphorylation was not affected by drug/glucose treatment were also excluded and iii) those residues (edges) whose phosphorylation status was not supported by our experimental data were eliminated. This filtering procedure yielded a much simpler network that was easier to analyze. For those proteins having multiple regulatory residues and whose phosphorylation was strongly changed in opposite direction, we considered only those sites quantified in a singly phosphorylated peptide (multiplicity =1).

Immunofluorescence microscopy

24 h following transfection Ins1e cells were fixed for 10 min in 4% paraformaldehyde (EM Sciences), washed with PBS, 0.5% Triton X-100, and permeabilized with blocking solution (0.1% Triton, 10% fetal calf serum) for 30 min. Coverslips were incubated in humidified chambers, in blocking solution, with anti-Flag TRITC antibody for 1 h at room temperature. Cells were rinsed in PBS and stained with 4′,6-diamidino-2-phenylindole in PBS, 0.1% Triton for 5 min at room temperature. Coverslips were mounted on slides as described and analyzed by indirect immunofluorescence microscopy.

Supplementary References

1. Cox J, Mann M. MaxQuant enables high peptide identification rates, individualized p.p.b.-range mass accuracies and proteome-wide protein quantification. *Nature biotechnology* **26**, 1367-1372 (2008).

2. Cox J, Neuhauser N, Michalski A, Scheltema RA, Olsen JV, Mann M. Andromeda: a peptide search engine integrated into the MaxQuant environment. *Journal of proteome research* **10**, 1794-1805 (2011).

3. Cox J, Hein MY, Luber CA, Paron I, Nagaraj N, Mann M. Accurate proteome-wide label-free quantification by delayed normalization and maximal peptide ratio extraction, termed MaxLFQ. *Molecular & cellular proteomics : MCP* **13**, 2513-2526 (2014).

4. Geiger T, Wehner A, Schaab C, Cox J, Mann M. Comparative Proteomic Analysis of Eleven Common Cell Lines Reveals Ubiquitous but Varying Expression of Most Proteins. *Molecular & Cellular Proteomics* **11**, (2012).

5. Tyanova S*, et al.* The Perseus computational platform for comprehensive analysis of (prote)omics data. *Nature methods*, (2016).

6. Cox J, Mann M. 1D and 2D annotation enrichment: a statistical method integrating quantitative proteomics with complementary high-throughput data. *BMC bioinformatics* **13 Suppl 16**, S12 (2012).

7. Sacco F*, et al.* Deep proteomics of breast cancer cells reveals that metformin rewires signaling networks away from a pro-growth state. *Cell Systems* **3**, 2-13 (2016).

8. Hornbeck PV, Zhang B, Murray B, Kornhauser JM, Latham V, Skrzypek E. PhosphoSitePlus, 2014: mutations, PTMs and recalibrations. *Nucleic acids research* **43**, D512-520 (2015).
